# Effect of PTTG on endogenous gene expression in HEK 293 cells

**DOI:** 10.1186/1471-2164-10-577

**Published:** 2009-12-03

**Authors:** Siva K Panguluri, Sham S Kakar

**Affiliations:** 1 Department of Physiology and Biophysics, James Graham Brown Cancer Center, University of Louisville, Louisville, KY 40202 USA; 2 Anatomical Sciences and Neurobiology, School of Medicine, University of Louisville, Louisville, KY 40202 USA

## Abstract

**Background:**

Pituitary tumor transforming gene (PTTG), also known as securin, is highly expressed in various tumors including pituitary, thyroid, colon, ovary, testis, lung, and breast. An overexpression of PTTG enhances cell proliferation, induces cellular transformation *in vitro*, and promotes tumor development in nude mice. PTTG also inhibits separation of sister chromatids leading to aneuploidy and genetic instability. A great amount of work has been undertaken to understand the biology of PTTG and its expression in various tumors. However, mechanisms by which PTTG mediates its tumorigenic function are not fully understood. To utilize this gene for cancer therapy, identification of the downstream signaling genes regulated by PTTG in mediation of its tumorigenic function is necessary. For this purpose, we expressed PTTG in human embryonic kidney (HEK293) cells that do not express PTTG and analyzed the downstream genes using microarray analysis.

**Results:**

A total of 22,277 genes printed on an Affymetrix HG-U133A 2.0 GeneChip™ array were screened with labeled cRNA prepared from HEK293 cells infected with adenovirus vector expressing PTTG cDNA (AdPTTG cDNA) and compared with labeled cRNA prepared from HEK293 cells infected with control adenovirus (control Ad) or adenovirus vector expressing GFP (AdGFP). Out of 22,277 genes, 71 genes were down-regulated and 35 genes were up-regulated with an FDR corrected p-value of ≤ 0.05 and a fold change of ≥2. Most of the altered genes identified are involved in the cell cycle and cell apoptosis; a few are involved in mRNA processing and nitrogen metabolism. Most of the up-regulated genes belong to the histone protein family.

**Conclusion:**

PTTG is a well-studied oncogene for its role in tumorigenesis. In addition to its importance in regulation of the cell cycle, this gene has also been recently shown to play a role in the induction of cell apoptosis. The microarray analysis in the present study demonstrated that PTTG may induce apoptosis by down-regulation of oncogenes such as v-Jun and v-maf and up-regulation of the histone family of genes.

## Background

Pituitary tumor transforming gene (PTTG) is a multifunctional protein involved in the cell cycle, cell proliferation, angiogenesis, metastasis, and other cellular functions. PTTG was initially isolated from rat pituitary tumor [1]. Based on its function in the inhibition of separation of sister chromatids, it was named securin [2]. Overexpression of PTTG was reported in many cancers including ovary, lung, testis, kidney, colon, thyroid, pituitary, liver, adrenal, breast, prostate, melanoma, leukemia, and lymphoma [3-15]. Many laboratories have explored this oncogene for its role in tumorigenesis since its identification (see reference [16] for review). Pei and Melmed [1] showed that overexpression of PTTG in NIH3T3 cells inhibits cell proliferation and induces cell transformation *in vitro*. In contrast, overexpression of PTTG in human embryonic kidney (HEK293) cells was shown to increase cell proliferation, induce cellular transformation *in vitro*, and promote tumor development in nude mice [7,17]. Ishikawa et al. [18] and McCabe et al. [19] showed PTTG's role in angiogenesis by coinciding its function in inducing the expression of basic fibroblast growth factor (bFGF) and vascular endothelial cell growth factor (VEGF). Heaney et al. [20] reported regulation of PTTG expression *in vitro *and *in vivo *by estrogen. Ishikawa et al. [18] demonstrated that the conditioned medium collected from NIH3T3 cells overexpressing human PTTG induced angiogenesis through up-regulation of bFGF both *in vitro *and *in vivo*. This information was further confirmed by Kim et al. [21] who showed regulation of angiogenic genes such as ID3 and TSP-1 by PTTG. Angiogenic gene ID3 has been shown to be up-regulated by VEGF [22], which is believed to play a critical role in cell proliferation and to be a precursor of endothelial cell recruitment [23,24]. Shibata et al. [25] showed the importance of PTTG in tumor metastasis and its correlation with the pathological stage, levels of pain, and extensive lymph node metastasis in esophageal cancer patients. Its expression levels were found to be correlated with a higher degree of tumor recurrence and tumor aggression in breast cancer as well as in squamous cell carcinoma [26,27]. In our previous studies, we [28] demonstrated the up-regulation of matrix metalloproteinase (MMP)-2 and its relationship in tumor angiogenesis and metastasis.

Role of PTTG in tumorigenesis was further confirmed by down-regulation of PTTG in tumors. Chen et al[29] down-regulated PTTG expression using full-length anti-PTTG complementary (c) DNA in SKOV3 and showed a significant reduction of cell proliferation and colony formation on soft agar compared to the non-transformed cells and bFGF protein levels. In this context, using PTTG siRNA to transfect an ovarian tumor cell line (A2780), El-Naggar et al. [30] showed a 50% reduction in cell proliferation and a 70% reduction in colony formation. In addition, these investigators demonstrated a reduced incidence of tumor development and tumor growth in nude mice injected with A2780 cells that constitutively expressed PTTG siRNA compared to cells expressing control siRNA. These results were confirmed by Cho-Rok et al. [31] using an adenovirus expression system expressing PTTG siRNA in a hapotoma cell line.

The molecular mechanisms by which PTTG achieves its functions remain unclear. Although a limited number of individual target genes in various cell lines have been identified, a large-scale profile of the target genes regulated by PTTG had not been undertaken at the time of this study. For this purpose, we explored the cDNA-microarray technology to identify the differentially expressed genes by PTTG. Most of the tumors and cancer cell lines overexpress PTTG; therefore, we selected HEK293 cells, which expresses a very low or undetectable level of endogenous PTTG. The change in expression of some genes by PTTG identified by cDNA microarray analysis was confirmed by quantitative real-time PCR (qRT-PCR).

## Methods

### Cell lines

HEK293 cells were propagated in Dulbecco's modified Eagle's medium (DMEM) supplemented with 10% fetal bovine serum (FBS) according to instructions from American Type Culture Collection (ATCC; Manassas, VA). Medium and serum were purchased from Invitrogen (Carlsbad, CA). Cells were grown under 5% CO_2 _at 37°C.

### Plasmid construct

The full length PTTG cDNA was sub-cloned into adenovirus shuttle vector pShuttle. Positive clones were sequenced to confirm the sequence and orientation of cDNA. The adenovirus expression system was generated and purified in association with the Gene Therapy Center, Virus Vector Core Facility, University of North Carolina at Chapel Hill.

### Expression of PTTG in HEK293 cells using the adenovirus expression system

HEK293 cells growing in log phase were trypsinized and plated on T-75 flasks. Cells were infected with adenovirus vector, adenovirus vector expressing GFP (AdGFP), or adenovirus vector expressing PTTG cDNA (AdPTTG cDNA) at variable multiplicities of infection (MOI). Infection of cells was carried out in serum-free DMEM medium for 2 h. After 2 h, the medium was replaced with regular growth medium and incubated at 37°C under 5% CO_2_. The optimum MOI was found to be 1:10 to provide a 90- to 95% level of infection. Experiments were performed in accordance with instructions from the University of Louisville Institutional Biosafety Committee.

### Isolation of total RNA

After 48 h of post-infection with adenovirus at a MOI of 1:10, medium was aspirated and cells were rinsed twice with phosphate-buffered saline (PBS). Cells were harvested by scraping with a rubber policeman and centrifuged at 5000 rpm for 5 min. The medium was discarded and 1 ml of Triazol reagent (Sigma-Aldrich, St. Louis, MO) was added to the cell pellet. The cells were homogenized and total RNA was purified as described previously [7]. Quality of RNA was examined on agarose gel electrophoresis and quantified by NanoDrop 1000 (Thermo Scientific, Waltham, MA). All treatments were performed in triplicate.

### Western blot analysis

Cells were cultured in complete growth media to 80-90% confluence. Cells were washed and collected in cold PBS and lysed with lysis buffer (10 mM Tris-HCl, pH 7.5; 150 mM NaCl; 1% Triton X-100; 1 mM EDTA). Protein concentration for each sample was assayed by Bradford Method (Bio-Rad Laboratories). Forty micrograms of protein from each sample was resolved on 15% SDS polyacrylamide gel and transferred to nitrocellulose membranes (GE Healthcare Biosciences, Piscataway, NJ). Membranes were blocked overnight with 5% non-fat milk/Tween (20 mM Tris/HCl, 150 mM NaCl, 0.1% Tween-20, pH 7.6) (TBST). Membranes were incubated with PTTG antiserum diluted at (1:1,500) [32] for 1 h at room temperature followed by 3 washes with TBST and then incubated with HRP-conjugated goat anti-rabbit secondary antibody diluted at 1:5,000. The immune complexes formed were detected by ECL reagents (GE Healthcare Biosciences). The blots were stripped and reprobed with β-actin antibody (Sigma-Aldrich, USA.) to examine equal loading.

### Microarray analysis

The microarray analysis was performed at the University of Louisville Microarray Core Facility according to instructions from Affymetrix (Santa Clara, CA). mRNA was converted into double stranded cDNA using a T7-oligo (dT) promoter primer sequence. The double-stranded cDNA was purified and served as a template in the subsequent *in vitro *transcription reactions. The *in vitro *transcription reactions were carried out in the presence of T7 RNA polymerase and a biotinylated nucleotide analog/ribonucleotide mix for cRNA amplification. The biotinylated cRNA was purified, fragmented, and used in the hybridization cocktail containing control oligonucleotide B2 and four control bacterial and phage cDNA (BioB, BioC, BioD, cre). The labeled cRNA was hybridized to GeneChip expression arrays Human U133A 2.0 (Affymetrix), containing 22,277 genes using the protocol described by Affymetrix and as described previously [33]. Alterations in RNA transcript levels (AdPTTG cDNA vs. adenovirus or adenovirus vs. AdGFP) were analyzed using Partek Genomics Suite 6.2 (Partek Inc., St. Louis, MO). Three different experiments were performed for statistical analysis.

### Data analysis

Data analysis was performed using Partek Genomics Suite 6.2 (Partek Inc., St. Louis, MO). The Affymetrix probe level signal values were summarized using the RMA algorithm. Statistically significant changed genes were identified by analysis of variance (ANOVA) with FDR-corrected p-values < 0.05. The contrast between adenovirus vs. AdGFP yielded no significantly different in genes based on these parameters. Two-way ANOVA tests were carried out to identify differentially expressed genes in the comparison of AdPTTG cDNA vs. adenovirus, taking treatment and batch effect for the triplicate sample processing into account. The genes that showed 2-fold induction or 2-fold suppression were transferred to separate up and down lists, respectively. The gene sets with an FDR corrected p-value of less than 0.05 were identified in these lists and Ingenuity Pathway Analysis software (Ingenuity Systems Inc., Redwood City, CA) was used to interpret the interactive pathway networks between the selected genes from the microarray data.

### qRT-PCR analysis

The genes that showed signal detection values more than 100, p-values less than 0.001, and a change in gene expression of more than 2-fold when compared to the control were selected to study their relative expressions by qRT-PCR using a MyiQ single color real-time PCR detection system (Bio-Rad Laboratories, Hercules, CA). qRT-PCR was performed in a total of 20 μl reaction volume containing 1 μl of cDNA, 1 μl each of forward- and reverse-specific primers (from 10 μM primer stock), 10 μl of supermix (Bio-Rad Laboratories), and 7 μl of nuclease-free water. All qRT-PCR reactions were performed using the following reaction conditions: initial denaturation at 95°C for 3 min followed by 45 cycles at 95°C for 10 s, 60°C for 20 s, and 72°C for 30 s. A fluorescence reading determined the extent of amplification at the end of each cycle.

Standard curves were obtained using a 10-fold serial dilution of pooled cDNA from all treatments. Expression of the human glyceraldehyde phosphate dehydrogenase (GAPDH) gene was used for normalization of data. For all candidate genes (Table 1), the quantities of the mRNA expression relative to GAPDH were obtained. All samples were measured in triplicate. The PCR efficiency and correlation coefficient values were taken into account before estimating the relative expression. Mean and standard errors for each treatment were obtained from all three replicates and each experiment was repeated at least twice.

**Table 1:** The primer sequences used for the validation of differentially expressed genes by PTTG overexpression through qRT-PCR.

S. No	Gene name	Forward Primer	Reverse primer
1	PTTG	5' gccttagatgggagatctca 3'	5' gctttaacagtcttctcagt 3'

2	v-jun	5' gcgtgcgctcttagagaaac 3'	5' ccgttgctggactggattat 3'

3	v-maf	5' cccgaccgaacagaagac 3'	5' gcttggtgatgatggtgatg 3'

4	H2be	5' ccgcaaagagagctactcca 3'	5' ggagctggtgtacttggtga 3'

5	H1c	5' agcgtagcggagtttctctg 3'	5' tagcgctcttcttcggagtt 3'

6	H2bo	5' gcagccgcaaagagacttac 3'	5' tggagctggtgtacttggtg 3'

7	H2ac	5' agaagaacaacagccgcatc 3'	5' gcttcttcgccttctttgg 3'

## Results and Discussion

### Adenovirus infection showed 90% efficiency

**Figure 1:**
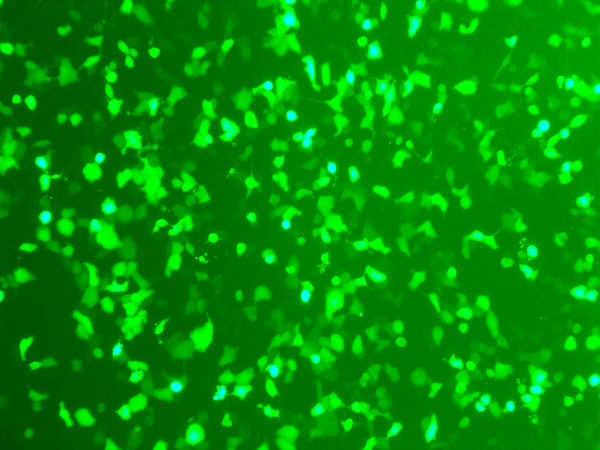
**Expression of AdGFP**. HEK293 cells were infected with AdGFP protein after 24 h post-infection.

**Figure 2:**
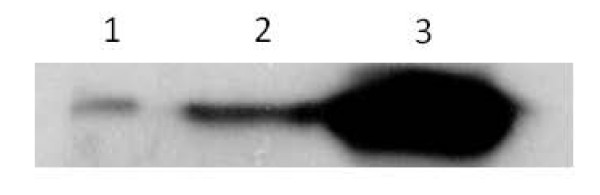
**Western blot analysis of PTTG protein expression in HEK293 cells infected with empty adenovirus, adenovirus with GFP and adenovirus with PTTG cDNA**.

The pShuttle vector carrying a full-length PTTG sequence was used in this study. A control adenovirus (control Ad) and AdGFP were used as controls. All three purified adenovirus constructs were used to infect HEK293 cells at variable MOIs to optimize a high level of infection. All the infected cells were examined for GPF expression after 24 h of infection (Fig. 1). The cells infected with AdGFP showed almost 95% level of infection at MOI of 1:10. Expression of PTTG was examined using Western blot analysis as described previously [32]. As shown in Fig. 2, a high level of expression of PTTG protein was observed in HEK293 cells infected with AdPTTG cDNA at 48 h post-infection.

### PTTG overexpression caused down-regulation of many genes

The microarray analysis of the human genome chip containing 22,277 genes showed a total of 106 genes differentially expressed in the experimental group, within an FDR corrected p-value ≤ 0.05 and fold change of ≥2 (Table 2). Among these 106 genes, 71 genes were down-regulated and 35 genes were up-regulated (additional file 1). The microarray data was deposited in public repository, ArrayExpress (http://www.ebi.ac.uk/arrayexpress, accession # E-MEXP-2449). The majority of the genes that were down-regulated belong to transcriptional factors, DNA binding proteins, and other interacting proteins involved in Wnt and Notch signaling pathways. Among the up-regulated genes, majority of genes belong to the histone protein family. Seven genes with the lowest p-values (≤ 0.0005) and high fold change (3 to 20) including PTTG, v-Jun and v-maf, H1c, H2be, H2bo, and H2ac were validated by qRT-PCR.

**Table 2:** The differentially expressed genes with different p-values in PTTG-expressing HEK293 cells by microarray analysis.

S. No	p-value	Fold change	Total genes	Up-regulated	Down-regulated
1	< 0.0001-0.0001	≥2	25	10	15

2	>0.0001-0.001	≥2	35	22	13

3	>0.001-0.01	≥2	22	2	20

4	>0.01-0.05	≥2	24	1	23

### v-Jun and Maf were down-regulated in PTTG-expressed HEK293 cells

**Figure 3:**
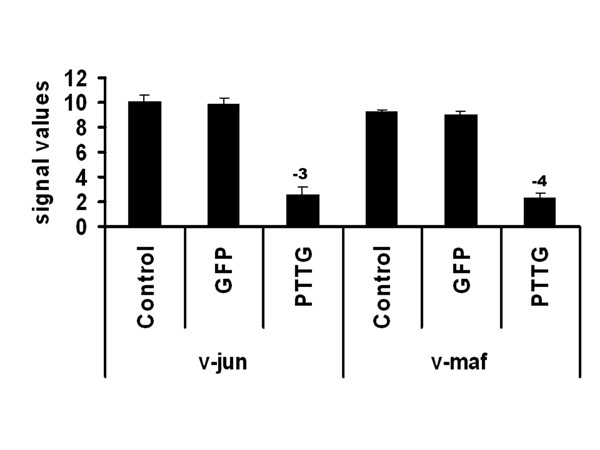
**PTTG down-regulated Jun and Maf expression**. A: Normalized expression of v-Jun and v-Maf in negative control, GFP control, and PTTG-overexpressed HEK293 cells by microarray analysis. The normalized expression values are mean ± SD (n = 3). The numbers above the bar represent the fold change of treatment with control group.

**Figure 4:**
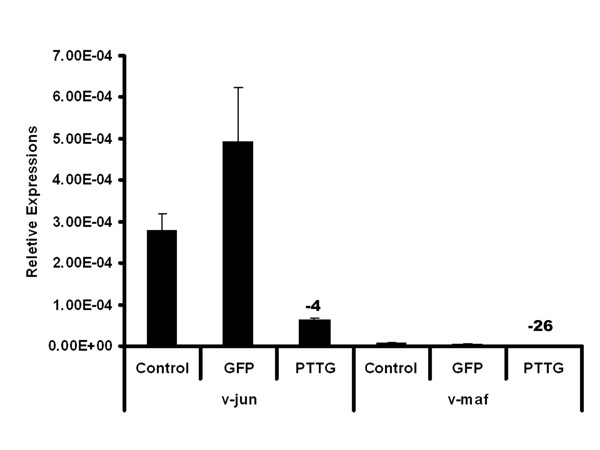
**PTTG down-regulated Jun and Maf expression**. B: The relative expression of v-Jun and v-Maf in negative control, GFP control, and PTTG-overexpressed HEK293 cells by qRT-PCR. The relative expression values are mean ± SD (n = 3). The numbers above the bar represent the fold change of treatment with control group.

The microarray analysis of HEK293 cells infected with AdPTTG cDNA showed significant down-regulation of the oncogene v-Jun. This gene was initially identified as the oncogenic factor of avian sarcoma virus 17 (ASV17) [34]. The cellular homologue of v-Jun is c-Jun, which is a predominant component of AP-1 transcriptional factors. The c-Jun oncogene can form homodimers and also forms heterodimers with Fos family members. This complex binds to TPA-response elements [34] and regulates a diverse range of biological functions ranging from cell growth and differentiation to stress signaling pathways [35]. The biochemical studies on c-Jun showed that this gene negatively regulates the association of p53 with p21CIP1 gene promoter [36]. Experiments by Maclaren [37] showed that the mRNA and protein levels of p21CIP1 were greatly reduced by v-Jun in chicken embryo fibroblasts (CEFs). They also found that the repression of p21CIP1 occurs by both p53-dependent and -independent mechanisms. The cell cycle regulator p21CIP1 is a cycle-dependent kinase inhibitor, which arrests the cell cycle throughout the G1/S phase. The present study showed down-regulation of v-Jun by overexpression of PTTG in HEK293 cells in both microarray and qRT-PCR analyses (Fig. 3 and 4). The microarray data showed a down-regulation of this gene by 3.86-fold. The qRT-PCR analysis of this gene showed 4.2-fold down-regulation, which is in directional correspondence with the microarray data. From these observations, we believe that overexpression of PTTG may induce p21CIP1 by down-regulating its inhibitor v-Jun. There are also reports that the p21CIP1 promoter is activated by a tumor-suppressor gene p53 [38]. Our microarray data also showed a slight up-regulation of p53 by PTTG-over expression (data not shown). In our previous studies, we showed up-regulation of p53 transcription by PTTG [17]. There is evidence that PTTG can induce expression of p21CIP1 [39], which is also slightly up-regulated in our microarray data (results not shown).

**Figure 5:**
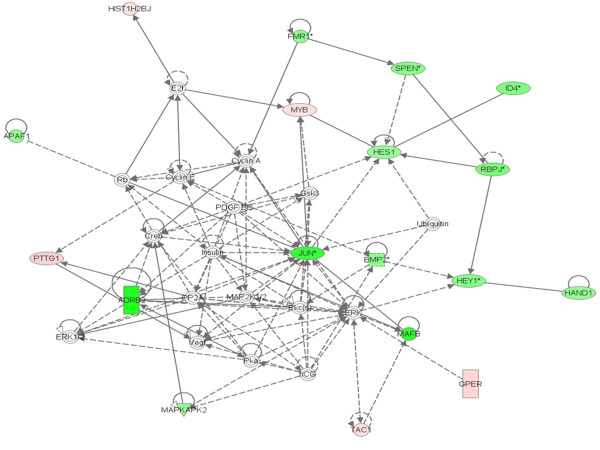
**The Ingenuity pathway analysis of differentially expressed genes interlinked with the v-Jun regulation by PTTG**.

**Figure 6:**
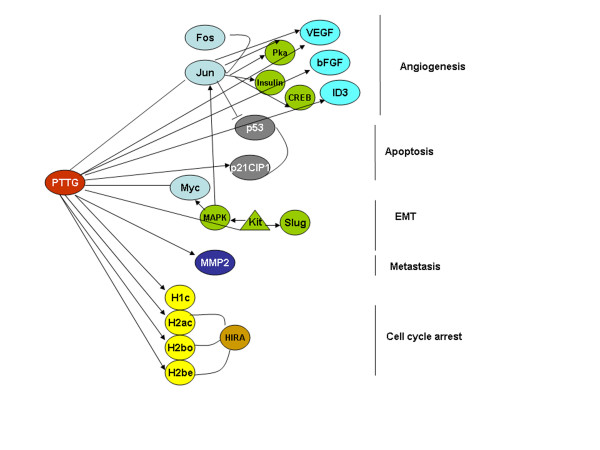
**Schematic diagram of PTTG's role in different pathways**.

The Ingenuity Pathway analysis of the selected genes from our microarray data showed that the oncogene Jun is involved in the expression of hairy and enhancer of split 1 (Hes1) in Rat1a cells [40], which is reported to interact with a dominant negative helix-loop-helix protein called inhibitor of DNA binding 4 (ID4) [41]. Our microarray data also showed the down-regulation of these two genes (Hes1 and ID4), which might be the downstream targets of Jun. In addition to these genes, Jun proteins are involved in regulation of many genes such as cAMP, cAMP-dependent protein kinase (Pka), insulin, cAMP response element binding protein (CREB), VEGF, MAPK, and ERK transcriptional factors (Fig. 5). The Ingenuity pathway also showed that the PTTG1 induces expression of human VEGF mRNA [42], whereas Jun represses VEGF secretion [43]. Our previous studies on xenograft models of HEK293 cells overexpressing PTTG1 by transiently in nude mice induced the secretion of bFGF, VEGF and IL-8 protein levels significantly over the mice injected with normal HEK293 cells [44]. We also identified the induced expressions of VEGF, bFGF and IL-8 in the HEK293 cells transiently transfected with PTTG1 cDNA *in vitro*. Similar findings were also observed by other investigators where PTTG was found to induce cell proliferation via VEGF, and PTTG expression levels were always observed with the induced levels of FGF and VEGF [45,46]. But the microarray analysis of HEK293 cells overxpressing PTTG in the present study did not show changes in VEGF, bFGF and IL-8 transcripts significantly within the selected stringency. Although we observed a slight increase in these three transcripts (≤1.5-fold) they are filtered in the selected stringent conditions (p-value ≤ 0.05 and ≥2-fold). As we know that the cDNA-microarray can only provide approximations of gene transcripts, which is further associated with some experimental noises that detracts the experiment ability, we expect loss or gain of signal intensities. Also the previous studies showed the expression of VEGF and FGF by PTTG in transient transfection experiments, which express moderate levels of PTTG protein. Whereas in the present study we utilized adenoviral mediated expression of PTTG, which yields higher levels of PTTG protein expressions, which could be an additional possibility of variation in the experimental data. PTTG being an important protein involved in various cellular pathways, from the present study we also observed that the influence of PTTG on various cell signaling is more of quantitative than generic. From these observations, it may be possible that PTTG is involved in angiogenesis both by inducing expression of angiogenic factors such as VEGF and bFGF as well as by the down regulation of transcriptional factors that repress the secretion of VEGF (Fig. 6).

The present study also showed the down-regulation of another leucine zipper-bearing transcriptional factor, v-Maf, by 4-fold in microarray data, which is consistent with qRT-PCR analysis (Fig. 3 and 4). The recent findings also suggest that mutated Jun, which lacks a transactivation domain, represses cell transformation not only by itself, but also by another oncogene, Maf [47]. From their studies, these investigators suggest that these two oncogenes share downstream targets and induce cell transformation through similar genes. Our studies showed a correlation with these findings as both of these genes down-regulated in microarray as well as in qRT-PCR. The ingenuity pathway analysis showed that members of Maf gene transcriptional factors regulate insulin gene transcription in islet cells [48]. In turn, this was supported by the findings of Wang et al. [49], who showed that PTTG knockout mice severely impair glucose homeostasis leading to diabetes during late adulthood due to impaired beta cell proliferation. From these observations, it is evident that PTTG has its role in the cell cycle and tumorigenesis, but also is involved in maintaining glucose homeostasis.

**Figure 7:**
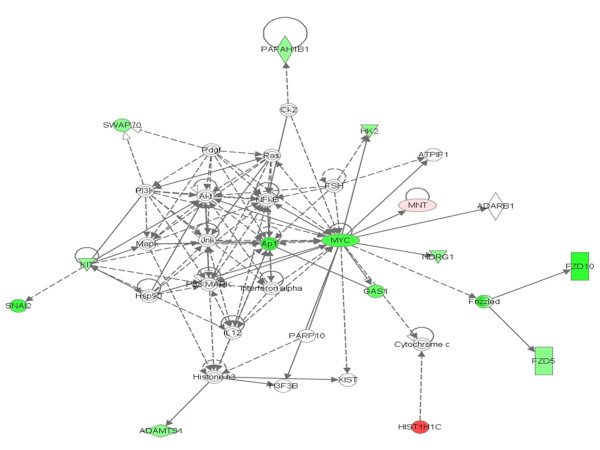
**The Ingenuity pathway analysis of differentially expressed genes interlinked with the v-Maf regulation by PTTG**.

In addition to these relations, the Ingenuity pathway analysis (Fig. 7) also showed that the oncogene v-Myc is regulated by MAPK, which in turn is regulated by c-Kit which regulates human Slug (Snail 2) [50,51]. It was also known that the oncogene Jun can be regulated by MAPK and ERK[52]. Our microarray data also showed that along with Jun and Myc, c-Kit and Slug were also down-regulated. Slug (Snail 2) is a member of the snail family of transcriptional factors, which is found to have a major role in epithelial-mesenchymal transition (EMT). The recent experiments on MDCK cell lines with Slug overexpression showed that this gene promotes EMT by inducing discoid domain receptor (DDR)-2 expression [53]. Also, the studies of Vitali et al. [54] showed that the knockout of Slug by RNAi facilitates apoptosis and inhibits invasive growth in neuroblastoma preclinical models. From these findings, it can be concluded that the overexpression of PTTG can induce apoptosis either by inducing expression of p21CIP1[39] or by inducting Bcl-2 expression [54].

### Histone family of genes was up-regulated by PTTG overexpression

**Figure 8:**
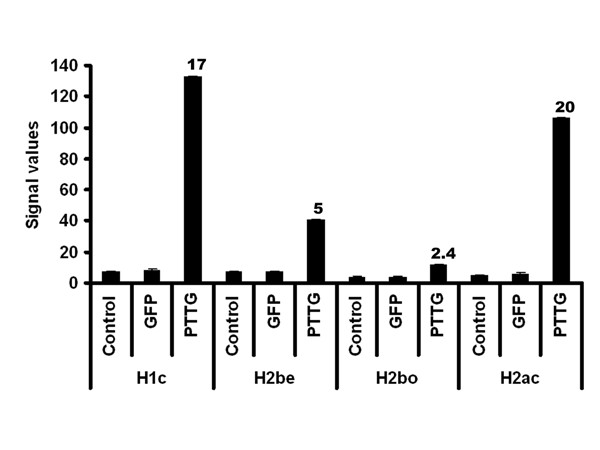
**PTTG up-regulates Histone gene expressions**. **A**: Normalized expressions of H2be, H2bo, H2ac, and Hic in negative control, GFP control, and PTTG-overexpressed HEK293 cells by microarray analysis. Normalized expression values are mean ± SD (n = 3). The numbers above the bar represent the fold change of treatment with control group.

**Figure 9:**
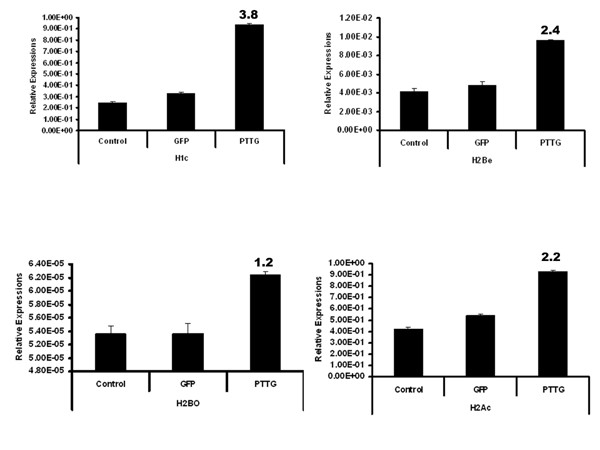
**PTTG up-regulates Histone gene expressions**. **B**: The relative expression of H2be, H2bo, H2ac, and Hic in negative control, GFP control, and PTTG-overexpressed HEK293 cells by qRT-PCR. The relative expression values are mean ± SD (n = 3). The numbers above the bar represent the fold change of treatment with control group.

**Figure 10:**
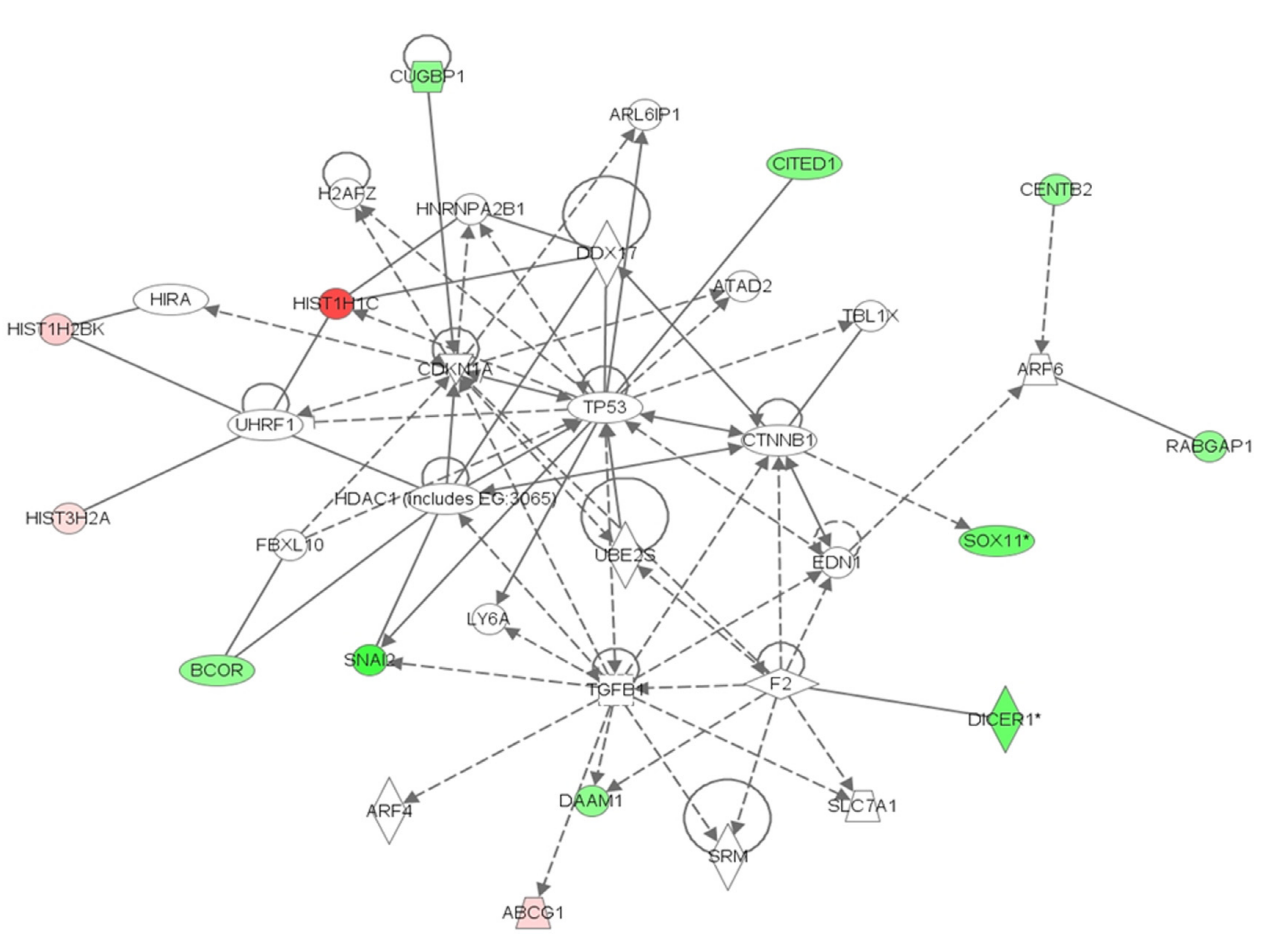
**The Ingenuity pathway analysis of differentially expressed genes interlinked with the Histone family gene regulation by PTTG**.

Long-term modifications in the chromatin may cause changes in gene expression and there by regulate many important cellular pathways. The fundamental unit of chromatin, nucleosome, is composed of 146 bps of genomic DNA wrapped by an octomer of the core histone proteins H2a, H2b, H3, and H4. Each class of histone proteins consists of several subtypes that are encoded by different genes. These genes show high similarity in sequence and are synthesized during the S phase of the cell cycle. The histone proteins in the nucleosome were also found to have a direct role in execution of apoptosis [55]. Histone proteins can be modified to allow DNA to unwind and permit transcriptional factor to bind and transactivate. In addition to this, some modifications to histones can inhibit transcriptional factor binding. For example gene activation can be associated with histone acetylation by histone acetyl transferase (HAT) [56], whereas histone deacetylation by histone deacetylase (HDAC) can suppress gene expression. Also it was evident from the studies that phosphorylation of histone is associated with activation of various immediate-early genes [57,58]. Also the previous studies showed that methylation of arginine and lysine residues can lead to gene activation and repression respectively [59,60]. Though we found up-regulation of these HAT and argentine methyltransferase in PTTG1 overexpressed HEK293 cells over normal/control cell line, due to high p-value (0.2 and 0.4 respectively) we did not validate them further by qRT-PCR analysis. The overexpression of PTTG in HEK 293 cells in the present study also showed up-regulation of the histone family of genes including H2bj, H1c, H2be, H2aa, H2bo, H2bh, H2bd, H2bk, H2a, H2am, H2ac, and H3D. Out of these histone genes, we have validated H2be, H1c, H2bo, and H2ac by qRT-PCR analysis. All four of these histone genes showed directional correspondence with the microarray data. The microarray and qRT-PCR analysis of H2be, H2bo, H2ac, and H1c (Fig. 8 and 9) showed up-regulation of these genes in PTTG-infected cells indicating their possible role in apoptosis. The ingenuity pathway analysis (Fig. 10) showed that H1c is a cytochrome c-releasing factor that appears in the cytoplasm in a p53-dependent manner after irradiation and activates downstream destruction programs [61]. The ingenuity pathway also showed that HIRA can bind with Histine H2 [62]. HIRA is a homologue of two cell cycle-regulated repressors of histone gene expression in *Saccharomyces cerevisiae*, whose expression blocks S-phase progression [63]. Although there is not much known on the role of PTTG in histone regulation, from the available literature it is evident that the overexpression of PTTG can induce apoptosis by up-regulating histone proteins. From these observations, we can conclude that PTTG either choosing cellular proliferation and tumorigenesis or p21CIP1-induced apoptosis depends on its expression in a quantitative manner. As we have previously used the adenovirus to infect cells, which resulted in high expression of the gene, the data confirmed the induction of apoptosis. However, in the case of lipid or chemical transfection methods, which can only yield up to 70% transfection, showed the induction of cell proliferation and tumorigenesis. This dual role of PTTG therefore seems to be quantitative, making exploration of this gene important for disease regulation.

## Conclusion

PTTG is a well-known oncogene, which is found to be expressed in many tumors and tumor cell lines. Existing evidence shows that this gene is involved in cell proliferation, migration, tumor invasion, and metastasis. In addition to these tumorigenic functions, this gene plays an important role in cell division. The recent findings of PTTG in inducing apoptosis [39] brings forth a new role for this gene in causing apoptosis rather than inducing cell proliferation and tumorigenesis. To identify the differentially expressed genes by PTTG overexpression in HEK293 cells, we have explored the oligonucleotide-microarray and qRT-PCR techniques. Our data clearly indicate the down-regulation of v-Maf and Jun and the up-regulation of many histone genes, which may explain the pathways by which PTTG induces apoptotic genes. In addition, the fate of the cell that overexpresses PTTG to choose either a tumorigenic or an apoptotic pathway may be based on its quantity of expression, which reveals this gene as playing dual roles.

## Competing interests

The authors declare that they have no competing interests.

## Authors' contributions

SKP performed QRT-PCR, part of data analysis and wrote the manuscript. SSK performed the viral infection, RNA isolation, chip-hybridization and edited the manuscript. Both authors read and approved the final manuscript.

## Supplementary Material

Additional file 1 **List of genes differentially regulated by PTTG in HEK 293 cells**. Table contains total number of genes which are up regulated or down regulated in HEK 293 cells infected with ad-PTTG compared to HEK 293 cells infected with ad-control. Click here for file
