# Supplementary material for: Effect of PTTG on endogenous gene expression in HEK 293 cells
Source: BMC Genomics. 2009 Dec 3;10:577. doi: 10.1186/1471-2164-10-577 (PMC2793268; doi:10.1186/1471-2164-10-577)
Supplement: Additional file 1 — List of genes differentially regulated by PTTG in HEK 293 cells. Table contains total number of genes which are up regulated or down regulated in HEK 293 cells infected with ad-PTTG compared to HEK 293 cells infected with ad-control. [file 1471-2164-10-577-S1.DOC]

Supplement table 1. List of genes differentially regulated by PTTG in HEK 293 cells

| **Affy ID** | **Gene Title** | **p-value** | **Fold Change** |
| --- | --- | --- | --- |
| 213139_at | snail homolog 2 (Drosophila) | 1.65E-07 | -3.58522 |
| 218839_at | hairy/enhancer-of-split related with YRPW motif 1 | 1.68E-06 | -2.3686 |
| 219764_at | frizzled homolog 10 (Drosophila) | 2.51E-06 | -3.95513 |
| 203554_x_at | pituitary tumor-transforming 1 | 2.89E-06 | 4.38588 |
| 44783_s_at | hairy/enhancer-of-split related with YRPW motif 1 | 3.34E-06 | -2.09362 |
| 214557_at | pituitary tumor-transforming 2 | 3.38E-06 | 13.8665 |
| 202934_at | hexokinase 2 | 8.57E-06 | -2.15497 |
| 209291_at | inhibitor of DNA binding 4, dominant negative helix-loop-helix protein | 1.23E-05 | -2.12624 |
| 208511_at | pituitary tumor-transforming 3 | 1.75E-05 | 10.6135 |
| 201466_s_at | jun oncogene | 2.12E-05 | -3.86766 |
| 206170_at | adrenergic, beta-2-, receptor, surface | 2.96E-05 | -4.27005 |
| 214522_x_at | histone cluster 1, H3d | 3.20E-05 | 3.383 |
| 201464_x_at | jun oncogene | 3.60E-05 | -2.49345 |
| 209771_x_at | CD24 molecule | 3.76E-05 | 2.03506 |
| 201465_s_at | jun oncogene | 4.01E-05 | -2.6427 |
| 205290_s_at | bone morphogenetic protein 2 | 4.02E-05 | -2.11913 |
| 208490_x_at | histone cluster 1, H2bf | 4.97E-05 | 5.2911 |
| 209292_at | Inhibitor of DNA binding 4, dominant negative helix-loop-helix protein | 5.18E-05 | -2.43889 |
| 204798_at | v-myb myeloblastosis viral oncogene homolog (avian) | 5.41E-05 | 2.25793 |
| 221582_at | histone cluster 3, H2a | 6.13E-05 | 3.17802 |
| 221743_at | CUG triplet repeat, RNA binding protein 1 | 6.53E-05 | -2.13188 |
| 204457_s_at | growth arrest-specific 1 | 7.43E-05 | -2.88552 |
| 214290_s_at | histone cluster 2, H2aa3 /// histone cluster 2, H2aa4 | 7.57E-05 | 11.0202 |
| 218559_s_at | v-maf musculoaponeurotic fibrosarcoma oncogene homolog B (avian) | 8.07E-05 | -4.00909 |
| 218280_x_at | histone cluster 2, H2aa3 /// histone cluster 2, H2aa4 | 9.73E-05 | 10.8389 |
| 206110_at | histone cluster 1, H3h | 0.000125448 | 8.00716 |
| 222162_s_at | ADAM metallopeptidase with thrombospondin type 1 motif, 1 | 0.000132118 | -2.21934 |
| 208180_s_at | histone cluster 1, H4h | 0.00013455 | 3.35942 |
| 209398_at | histone cluster 1, H1c | 0.000137101 | 17.7826 |
| 208527_x_at | histone cluster 1, H2be | 0.000138273 | 5.50506 |
| 208523_x_at | histone cluster 1, H2bi | 0.000148127 | 4.23292 |
| 208546_x_at | histone cluster 1, H2bh | 0.000156095 | 6.52747 |
| 219433_at | BCL6 co-repressor | 0.000156257 | -2.052 |
| 207046_at | histone cluster 2, H4a /// histone cluster 2, H4b | 0.000159328 | 3.28037 |
| 202708_s_at | histone cluster 2, H2be | 0.000160063 | 14.3925 |
| 222067_x_at | histone cluster 1, H2bd | 0.000160924 | 5.78925 |
| 202431_s_at | v-myc myelocytomatosis viral oncogene homolog (avian) | 0.000165935 | -3.45379 |
| 209911_x_at | histone cluster 1, H2bd | 0.000167692 | 10.7173 |
| 209301_at | carbonic anhydrase II | 0.000178408 | -2.15139 |
| 204567_s_at | ATP-binding cassette, sub-family G (WHITE), member 1 | 0.000179538 | 4.07493 |
| 213931_at | inhibitor of DNA binding 2, dominant negative helix-loop-helix protein /// inhib | 0.000180747 | -2.34504 |
| 202386_s_at | KIAA0430 | 0.000180945 | -2.2597 |
| 207156_at | histone cluster 1, H2ag | 0.000187179 | 10.7126 |
| 214455_at | histone cluster 1, H2bg /// histone cluster 1, H2bc | 0.000208423 | 6.54565 |
| 208579_x_at | H2B histone family, member S | 0.000209317 | 6.28002 |
| 209198_s_at | synaptotagmin XI | 0.000239937 | 2.60274 |
| 212313_at | CHMP family, member 7 | 0.000251022 | -2.21848 |
| 206552_s_at | tachykinin, precursor 1 (substance K, substance P, neurokinin 1, neurokinin 2, n | 0.000286946 | 2.30123 |
| 205289_at | bone morphogenetic protein 2 | 0.000303167 | -2.26192 |
| 200632_s_at | N-myc downstream regulated gene 1 | 0.000375801 | -2.14958 |
| 220138_at | heart and neural crest derivatives expressed 1 | 0.000377835 | -2.08077 |
| 214472_at | histone cluster 1, H3d | 0.000463089 | 4.93375 |
| 214540_at | histone cluster 1, H2bo | 0.000469653 | 3.10086 |
| 214481_at | Histone cluster 1, H2am | 0.00050498 | 2.88766 |
| 215071_s_at | histone cluster 1, H2ac | 0.000510272 | 20.1498 |
| 204206_at | MAX binding protein | 0.000547646 | 2.26825 |
| 209521_s_at | angiomotin | 0.000594845 | -2.88628 |
| 209806_at | histone cluster 1, H2bk | 0.000622015 | 4.86385 |
| 200799_at | heat shock 70kDa protein 1A | 0.000644396 | -3.59249 |
| 213116_at | NIMA (never in mitosis gene a)-related kinase 3 | 0.000647863 | -2.58215 |
| 222227_at | --- | 0.00103278 | 4.07794 |
| 209293_x_at | inhibitor of DNA binding 4, dominant negative helix-loop-helix protein | 0.00157294 | -2.08579 |
| 204866_at | PHD finger protein 16 | 0.00160591 | -2.65422 |
| 214502_at | histone cluster 1, H2bj | 0.00170781 | 3.04323 |
| 211089_s_at | NIMA (never in mitosis gene a)-related kinase 3 | 0.00204223 | -2.28397 |
| 203394_s_at | hairy and enhancer of split 1, (Drosophila) | 0.00221195 | -2.01143 |
| 207144_s_at | Cbp/p300-interacting transactivator, with Glu/Asp-rich carboxy-terminal domain, | 0.00232762 | -2.3447 |
| 201997_s_at | spen homolog, transcriptional regulator (Drosophila) | 0.00240513 | -2.32776 |
| 215128_at | CDNA FLJ11682 fis, clone HEMBA1004880 | 0.00294914 | -2.25117 |
| 214948_s_at | TATA element modulatory factor 1 | 0.00326285 | -2.81839 |
| 207781_s_at | zinc finger protein 711 | 0.00389047 | -2.04415 |
| 213281_at | Jun oncogene | 0.00482477 | -2.00864 |
| 204913_s_at | SRY (sex determining region Y)-box 11 | 0.00505387 | -2.48154 |
| 214949_at | CDNA FLJ31919 fis, clone NT2RP7004964 | 0.0053862 | -2.94789 |
| 216060_s_at | dishevelled associated activator of morphogenesis 1 | 0.00593811 | -2.13674 |
| 216361_s_at | MYST histone acetyltransferase (monocytic leukemia) 3 | 0.0059859 | -2.11249 |
| 204859_s_at | apoptotic peptidase activating factor 1 | 0.00612863 | -2.07116 |
| 213123_at | microfibrillar-associated protein 3 | 0.00710101 | -2.17071 |
| 211974_x_at | recombination signal binding protein for immunoglobulin kappa J region | 0.00771879 | -2.60633 |
| 219779_at | zinc finger homeobox 4 | 0.00811452 | -2.2118 |
| 219297_at | WD repeat domain 44 | 0.00950148 | -2.59478 |
| 203543_s_at | kruppel-like factor 9 | 0.0095213 | -2.85423 |
| 204914_s_at | SRY (sex determining region Y)-box 11 | 0.0100522 | -2.80436 |
| 218279_s_at | histone cluster 2, H2aa3 | 0.0102562 | 2.2075 |
| 201996_s_at | spen homolog, transcriptional regulator (Drosophila) | 0.0107577 | -2.18771 |
| 221245_s_at | frizzled homolog 5 (Drosophila) | 0.0119806 | -2.20456 |
| 212476_at | centaurin, beta 2 | 0.012313 | -2.07683 |
| 201461_s_at | mitogen-activated protein kinase-activated protein kinase 2 | 0.0131799 | -2.01275 |
| 212888_at | dicer1, Dcr-1 homolog (Drosophila) | 0.0158937 | -2.62201 |
| 207785_s_at | recombination signal binding protein for immunoglobulin kappa J region | 0.0162663 | -2.58305 |
| 206061_s_at | dicer1, Dcr-1 homolog (Drosophila) | 0.0176947 | -2.85858 |
| 200800_s_at | heat shock 70kDa protein 1A /// heat shock 70kDa protein 1B | 0.0176996 | -2.0471 |
| 203689_s_at | fragile X mental retardation 1 | 0.0209561 | -2.19451 |
| 209733_at | hypothetical protein LOC286440 | 0.0247722 | -2.27926 |
| 213229_at | dicer1, Dcr-1 homolog (Drosophila) | 0.0264143 | -2.53069 |
| 200816_s_at | platelet-activating factor acetylhydrolase, isoform Ib, alpha subunit 45kDa | 0.0267333 | -2.02579 |
| 215245_x_at | fragile X mental retardation 1 | 0.0324739 | -2.13946 |
| 213313_at | RAB GTPase activating protein 1 | 0.0332312 | -2.05143 |
| 219312_s_at | zinc finger and BTB domain containing 10 | 0.0359223 | -2.60828 |
| 209307_at | SWAP-70 protein | 0.0369236 | -2.21634 |
| 205051_s_at | v-kit Hardy-Zuckerman 4 feline sarcoma viral oncogene homolog | 0.038958 | -2.07989 |
| 201855_s_at | ATM/ATR-Substrate Chk2-Interacting Zn2+-finger protein | 0.049146 | -2.03411 |
| 212150_at | KIAA0143 protein | 0.0535425 | -2.18383 |
| 219631_at | low density lipoprotein-related protein 12 | 0.0604262 | -2.04904 |
| 213552_at | glucuronic acid epimerase | 0.0702648 | -2.04806 |
| 212149_at | KIAA0143 protein | 0.0768093 | -2.13148 |
